# Radioresistance of cancer cells, integrin αvβ3 and thyroid hormone

**DOI:** 10.18632/oncotarget.26434

**Published:** 2018-12-11

**Authors:** John T. Leith, Shaker A. Mousa, Aleck Hercbergs, Hung-Yun Lin, Paul J. Davis

**Affiliations:** ^1^ Rhode Island Nuclear Science Center, Narragansett, RI, USA; ^2^ Pharmaceutical Research Institute, Albany College of Pharmacy and Health Sciences, Rensselaer, NY, USA; ^3^ Department of Radiation Oncology, Cleveland Clinic, Cleveland, OH, USA; ^4^ Taipei Cancer Center, Taipei Medical University, Taipei, Taiwan; ^5^ PhD Program for Cancer Molecular Biology and Drug Discovery, College of Medical Science and Technology, Taipei Medical University, Taipei, Taiwan; ^6^ Traditional Herbal Medicine Research Center, Taipei Medical University, Taipei, Taiwan; ^7^ TMU Research Center of Cancer Translational Medicine, Taipei Medical University, Taipei, Taiwan; ^8^ Department of Medicine, Albany Medical College, Albany, NY, USA

**Keywords:** integrin open configuration, AKT, epithelial-mesenchymal transition (EMT), L-thyroxine (T_4_), STAT3

## Abstract

Radioresistance is a substantial barrier to success in cancer management. A number of molecular mechanisms support radioresistance. We have shown experimentally that the thyroid hormone analogue receptor on the extracellular domain of integrin αvβ3 may modulate the state of radiosensitivity of tumor cells. Specifically, tetraiodothyroacetic acid (tetrac), a derivative of L-thyroxine (T_4_), can reduce radioresistance in cancer cells. In this review, we list a number of intrinsic signal transduction molecules and other host factors that have been reported to support/induce radioresistance in cancer cells and that are also subject to control by T_4_ through actions primarily initiated at integrin αvβ3. Additional preclinical evidence is needed to support these radioresistance-relevant actions of thyroid hormone.

## INTRODUCTION

Recent reviews of the expression of radioresistance in cancer of the pancreas [1], gliomas [2], head and neck cancers [3], prostate cancer [4], urinary bladder cancer [5] and cancers of other organs [6, 7] emphasize the clinical importance of the state of radioresistance and the complexity of its molecular mechanisms. Radioresistance may be an inherent quality of a tumor or may emerge in the course of cancer management.

Spontaneous or medically induced hypothyroidism may beneficially alter the clinical behavior of a number of cancers, e.g., breast cancer [8], glioblastoma [9], head-and-neck tumors [10] and renal cell carcinoma [11]. Induction of the clinical state of euthyroid hypothyroxinemia—in which euthyroidism is maintained by administered 3,5,3′-triiodo-L-thyronine (T_3_) in the absence of host L-thyroxine (T_4_)—has also been shown to slow the course of end-stage carcinomas of various origins [12]. T_4_ in physiological concentrations *in vitro* has been shown to cause proliferation of a wide variety human cancer cells [13–16], whereas T_3_ in physiological concentrations does not appear to promote cancer cell proliferation *in vitro* [14]. Reports that T_3_ may stimulate tumor cell proliferation *in vitro* have relied upon high concentrations of the hormone [17, 18]. Clinically, circulating T_3_ may be quite low in cancer patients subject to the nonthyroidal illness syndrome [19].

The proliferative effects of T_4_ on cancer cells are initiated at a cell surface receptor for thyroid hormone on the extracellular domain of integrin αvβ3 [20, 21]. T_4_ is also anti-apoptotic via the receptor on αvβ3 [22] and supports tumor-relevant angiogenesis via this receptor [23]. The tumor-support actions of T_4_ at integrin αvβ3 on cancer cells are blocked by a derivative of T_4_, tetraiodothyroacetic acid (tetrac), either unmodified or reformulated as a nanoparticulate agent (Nano-diamino-tetrac (NDAT) or Nanotetrac) [20, 21], and these compounds by their anti-proliferative and pro-apoptotic actions [22] also reduce tumor xenograft size [24–29].

Importantly, tetrac has also been shown to radiosensitize glioma cells [30, 31]. Studies *in vitro* disclosed that this thyroid hormone derivative decreased repair of double-strand DNA breaks (DSBs) induced by radiation [31] and that tetrac itself may cause DSBs. Unmodified tetrac and Nanotetrac that act exclusively at integrin αvβ3 block the binding of T_4_ to the integrin and prevent the proliferative and anti-apoptotic actions of T_4_ initiated at this site [20, 21]. The observation that tetrac compounds acting exclusively at the integrin are capable of radiosensitizing tumor cells suggests that T_4_, acting at exactly the same site and displaced by tetrac, contributes to the phenomenon of radioresistance. We have shown that exposure of human cancer cells *in vitro* to X-irradiation rapidly activates αvβ3 [32] and that unmodified tetrac blocks this profound change in the physical state of the integrin. The silencing mediator for retinoid and thyroid hormone receptor (SMRT) is a nuclear corepressor protein that appears to be important to repair of DSBs that are radiation-induced [33], possibly implicating thyroid hormone in the promotion of this mechanism of radioresistance. The integrin has been implicated in regulation of radioresistance/radiosensitivity [34, 35].

Tumor irradiation *in vitro* has recently been shown to activate cancer cell integrin αvβ3 [32]. Activation of the extracellular domain of the integrin is an extended state and is induced within minutes. It appears to be associated with radioresistance, and this response to radiation is blocked by tetrac. It is thought that the change in physical state of the integrin contributes to radioresistance, perhaps by inducing more complex intercellular interactions and decreasing cell division. The radiosensitizing properties of tetrac may include this action on the state of integrin αvβ3.

What is clear from the actions of tetrac formulations initiated at integrin αvβ3 is that iodothyronine receptor site on the integrin controls radiosensitivity-radioresistance, and that tetrac molecules acting at this site control a substantial number of signaling pathways that contribute to the radiation sensitivity status of the tumor cells expressing the integrin. We will in brief review here a) the actions of tetrac—and, in some cases, thyroid hormone—on cancer cell radiosensitivity and b) the effects of Nanotetrac on the signaling pathways relevant to the radiation sensitivity state of tumor cells.

## MECHANISMS THAT MAY CONTRIBUTE TO DEVELOPMENT OF RADIORESISTANCE

### Tetrac and tumor radiosensitivity

As noted above, tetrac has been shown to radiosensitize murine glioma GL261 cells [30] and human glioblastoma U87MG cells [31], as well as to restore radiosensitivity to resistant human basal cell carcinoma (TE.354.T) cells [36]. In the studies of U87MG cells, exposure of cells *in vitro* for 1 hour prior to radiation resulted in a more than 70% reduction in repair of DSBs induced by X-irradiation. The molecular basis of this action of unmodified tetrac on DNA repair is not established. The anticancer actions of unmodified tetrac and Nanotetrac are initiated exclusively at plasma membrane integrin αvβ3, but many effects of the agent are expressed downstream in specific gene expression or altered activities of nuclear co-repressor and co-activator proteins [21, 23]. The integrin is expressed generously by cancer cells but not by normal cells; hence, tetrac is unlikely to make nonmalignant cells more radiosensitive.

### Signaling pathways involved in regulation of radiosensitivity and known effects of thyroid hormone analogues on these pathways

#### AKT

The phosphatidylinositol 3-kinase (PI-3K)/AKT (protein kinase B)/mTOR signal transduction pathway is frequently disordered in cancer cells and its excessive activity contributes to tumor cell proliferation and radioresistance [37, 38]. The latter is a function of enhanced repair of DSBs that are induced by radiation [39]. Although it has been stated that it is unclear how AKT may be stimulated in cancer cells in the setting of radiation so that radioresistance is generated [2], we would point out that physiological levels of thyroid hormone as T_4_ activate the AKT pathway via the cell surface thyroid hormone receptor on integrin αvβ3 [20, 21]. We propose that endogenous T_4_ in the intact organism—preclinically in animal models or in the clinical setting—is a mechanism of support in tumor cells for AKT-dependent DSB repair.

#### STAT3

Among its multiple contributions to intracellular signaling in nonmalignant cells [40] and cancer cells, signal transducer and activator of transcription 3 (STAT3) is known when activated to promote radioresistance in a variety of tumors [7, 41], including gliomas and lung cancer. We showed that T_4_ nongenomically activates STAT3, where activation is defined as specific tyrosine phosphorylation of the protein and subsequent nuclear translocation of the STAT [42]. T_4_ at physiological free concentrations also was found nongenomically to potentiate induction by epidermal growth factor (EGF) of c-Fos expression, and c-Fos has subsequently been found to promote cancer cell radioresistance in glioma cells [43]. Thus, one or more STAT3-dependent mechanisms exist by which thyroid hormone as T_4_ may contribute to tumor radioresistance.

#### Wnt/β-catenin

A third signaling pathway that has been implicated in the induction of the radioresistant state in tumor cells is that involving Wnt/β-catenin. The pathway is involved in stem cell biology and is also an index of tumor cell aggressiveness [44–46]. In several models that enabled study of radioresistance development in glioblastoma cells, frankly increased levels of β-catenin correlated with radioresistance [47]. Nuclear uptake of β-catenin occurs in radiated glioblastoma cells and accompanies tumor aggressiveness that is reversed by inhibition of this signaling pathway [48]. T_4_ promotes β-catenin-dependent proliferation of colorectal cancer cells [49]. Inhibition of nongenomic T_4_-initiated actions at integrin αvβ3 with the T_4_ analogue, tetrac, increases tumor cell nucleus accumulation of Cby1, a protein inhibitor of nuclear actions of β-catenin and also downregulates nuclear accumulation of the catenin [50]. Such results are consistent with the function of this pathway as another mediator of the actions of T_4_ to induce the radioresistant state.

### Epithelial-mesenchymal transition (EMT) in conjunction with radioresistance

Radiation exposure of cancer cells may support cell aggressiveness via the EMT process [6]. For example, in an esophageal cancer cell line in which radioresistance was induced, the development of radioresistance was coupled with AKT-induced EMT [51]. Physiological levels of T_4_ act at integrin αvβ3 to promote EMT in ovarian cancer cells [52], and this may be an ancillary mechanism by which the hormone contributes to radioresistance, as well as to cancer cell invasiveness.

### Hypoxia

Cancer cells are significantly more radioresistant under hypoxic conditions [2, 6]. The resistance is at least in part due to generation of hypoxia-inducible factor 1α (HIF-1α) [2, 6] that stabilizes cells subjected to the hypoxic state. The molecular basis of the generation of radioresistance by HIF-1α is not yet clear. A number of studies have shown that thyroid hormone can stimulate transcription of HIF-1α [53–55]. However, supraphysiological amounts of T_3_ appear to be required at the hormone receptor on integrin αvβ3 in order to stimulate HIF-1α gene expression [53] and this may also be the case at the nuclear thyroid hormone receptor (TR) [54]. Further, hypoxic conditions may activate deiodinase 3 (DIO3, D3) and lead to deiodination and inactivation of T_3_ [56]. T_4_ does not affect HIF-1α transcription at integrin αvβ3 [53]. We can conclude that it is unclear whether actions of thyroid hormone on HIF-1α can be relevant to pathogenesis of radioresistance.

### microRNAs

A substantial panel of miRNAs has been identified that modulates radiosensitivity of cancer cells [2–4]. Relatively little information is available on thyroid hormone-specific miRNA abundance and cancer and radiosensitivity. Actions of thyroid hormone on miRNA-21 abundance and activity have been studied in basal cell carcinoma cells [57], and this miRNA is known to be associated with radioresistance and cell proliferation in glioma cells [2]. miRNA-21 is downregulated by T_3_ in basal cells [57], a potentially desirable effect that is interestingly attenuated by concomitant increase in D3 generated by the hormone. Recent clinical experience with euthyroid hypothyroxinemia [12] indicates that T_3_ does not stimulate growth of a variety of solid tumors, including glioblastoma. In this setting, the action of T_3_ on D3 is insufficient to block the tumor response to triiodothyronine. Additional information is needed on the possible actions of thyroid hormones on amounts of tumor-specific miRNAs and radiosensitivity.

In addition to the factors discussed in this review that are relevant to tumor cell radiosensitivity, the Hedgehog pathway, Notch, P53 and other cell mechanisms may condition the state of radioresistance [1, 2, 6, 58]. Certain of these factors may also be controlled by thyroid hormone via its receptor on integrin αvβ3, e.g., P53, whose activation/phosphorylation is inhibited by T_4_ [22], supporting anti-apoptosis in cells exposed to thyroid hormone. But interpreting changes in P53 behavior and apoptosis across cell lines [59] and in response to radiation [60, 61] has been shown to be complex.

## IMPLICATION OF INVOLVEMENT OF THYROID HORMONE AND INTEGRIN αVβ3 IN RADIOSENSITIVITY STATE ON CANCER CELLS

### Circulating T_4_ levels during periods of radiotherapy of cancer in euthyroid patients

**Figure 1:**
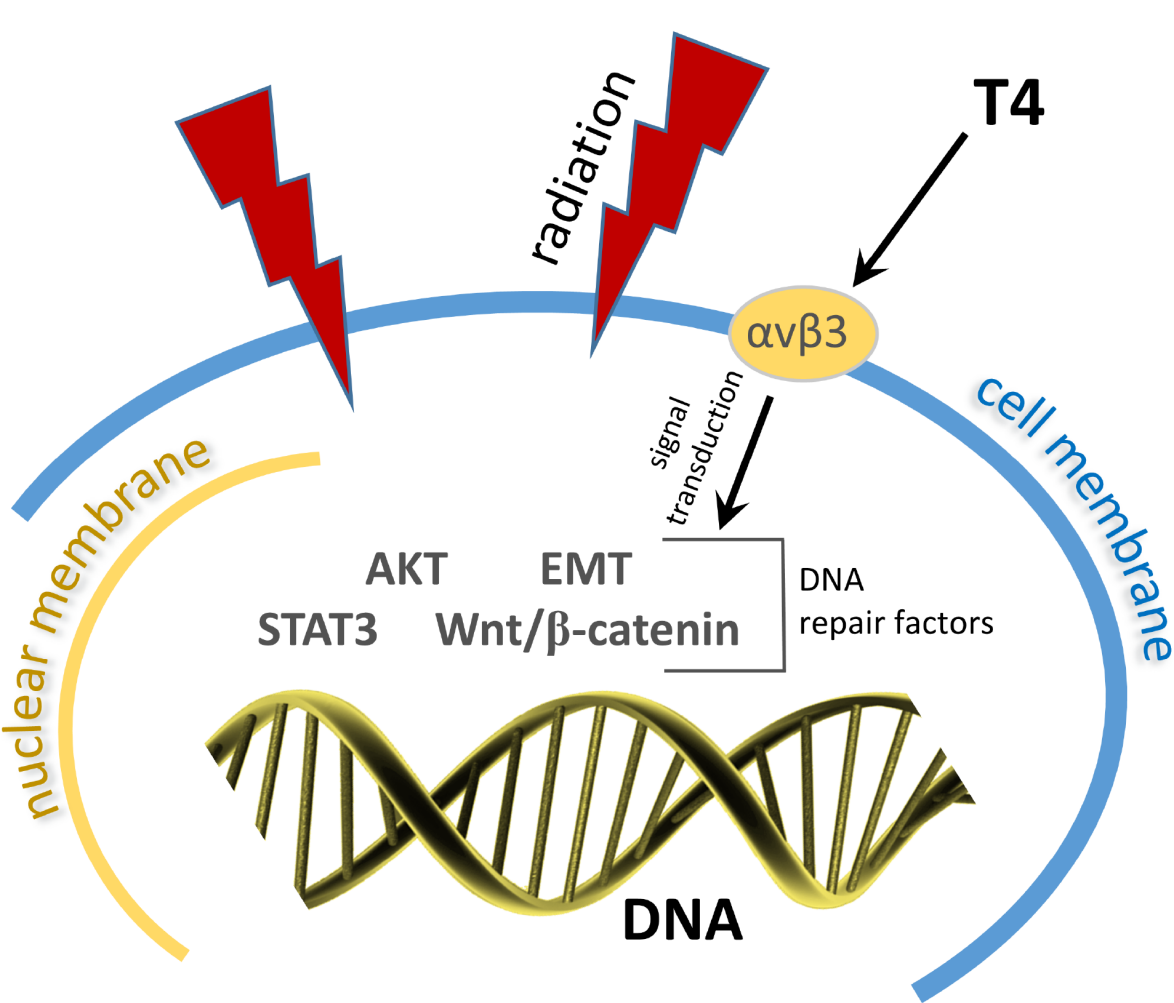
Radiation induces double-strand DNA breaks By several signaling pathways T4 acts at the tumor cell plasma membrane to activate multiple DNA repair factors (AKT, EMT, STAT3, Wnt/β-catenin, and possibly others).

Evidence discussed above supports the possibility that the thyroid hormone analogue receptor on integrin αvβ3 supports the expression of radioresistance by cancer cells. From these reports, it is the action of T_4_, rather than that of T_3_, that appears to be particularly relevant to the radiosensitivity state of the tumor cell (Figure 1). This possibility deserves additional preclinical investigation that compares tumor cell or xenograft sensitivity to X-irradiation in the presence and absence of T_4_ and T_3_, evaluating these forms of thyroid hormone individually.

There may be existing clinical settings in which a possible contribution of T_4_ to radioresistance may be assessed. Patients with advanced cancers who are managed palliatively with euthyroid hypothyroxinemia are a clinical population sometimes subjected to radiation for relief of pain, and it may be useful to evaluate the effectiveness of such radiation in term of analgesia, but also in terms of tumor size response to radiotherapy when a history of radioresistance is present. In addition, in cancer patients with hypothyroidism who may be candidates for radiotherapy, thyroid hormone replacement may be achieved with T_3,_ and studies are needed that are designed to assess the possibility that radiosensitivity—when radiation therapy is indicated—can be conditioned by T_4_. Thus, it may be useful to determine whether differences in radiosensitivity in tumor patients can be in part conditioned by whether circulating endogenous T_4_ levels are in the upper or lower quartile of the normal range.

Finally, tyrosine kinase inhibitor (TKI) anticancer drugs may induce thyroiditis that leads to usually transient hyperthyroidism and then to hypothyroidism [62, 63]. We would propose that radiotherapy should be avoided during a period of hyperthyroidism that is caused by TKI therapy.

## CONCLUSIONS

In this review, we have examined selected host factors that at the molecular level mediate the development of radioresistance in cancer cells. We have shown that thyroid hormone, particularly as T_4_, can activate these factors and, therefore, that T_4_ may support the emergence of radioresistance. We have preliminary *in vitro* evidence that T_4_ may reduce the effectiveness of X-irradiation in tumor cells and we recommend that additional preclinical evidence be sought that tests this possibility. Tetrac, an anti-T_4_ agent that acts at integrin αvβ3, has been shown to restore radiosensitivity in tumor cells. There are existing clinical settings in which the tumor radiosensitivity in the presence of T_4_ may be compared with that in the presence of T_3_ to test the possibility that T_4_ is a clinically relevant inducer of radioresistance.
